# No association between green tea and prostate cancer risk in Japanese men: the Ohsaki Cohort Study

**DOI:** 10.1038/sj.bjc.6603230

**Published:** 2006-06-27

**Authors:** N Kikuchi, K Ohmori, T Shimazu, N Nakaya, S Kuriyama, Y Nishino, Y Tsubono, I Tsuji

**Affiliations:** 1 Division of Epidemiology, Department of Public Health and Forensic Medicine, Tohoku University Graduate School of Medicine, 2-1 Seiryo-machi, Aoba-ku, Sendai, Miyagi 980-8575, Japan; 2 Department of Epidemiology, Miyagi Cancer Center, Natori 981-1293, Japan; 3 Division of Health Policy, Tohoku University School of Public Policy, Sendai 980-8576, Japan

**Keywords:** green tea, prostate cancer, prospective cohort study

## Abstract

In a prospective study of 19 561 Japanese men, green-tea intake was not associated with a lower risk of prostate cancer (110 cases), the multivariate hazard ratio for men drinking ≥5 cups compared with <1 cup per day being 0.85 (95% confidence interval 0.50–1.43, trend *P*=0.81).

Although laboratory studies have suggested a protective effect of green-tea polyphenols against development of prostate cancer in animal models (Gupta *et al*, 1999; Saleem *et al*, 2003), few epidemiological studies have examined the association. A case–control study in China found that green-tea intake was associated with a lower risk of prostate cancer (Jian *et al*, 2004), whereas a prospective study of Japanese Americans in Hawaii and a case–control study in Japan found no such association (Severson *et al*, 1989; Sonoda *et al*, 2004). The age-standardised incidence of prostate cancer is low in Japan (12.7 per 100 000), being approximately one-tenth of that in the US (Parkin, 2002). Green-tea consumption per capita in Japan is the highest in the world (International Tea Committee, 2004). One reason for the low incidence of prostate cancer in Japan may be the high consumption of green tea. We therefore examined the association between green-tea consumption and prostate cancer incidence among men in the Ohsaki Cohort Study conducted in rural Japan.

## MATERIALS AND METHODS

The details of the Ohsaki Cohort Study have been described previously (Tsuji *et al*, 1999; Anzai *et al*, 2005). Briefly, this prospective cohort study was started in 1994 and included 26 481 men aged 40–79 years living in 14 municipalities of Miyagi Prefecture (95% response rate) (Anzai *et al*, 2005). The study used a self-administered questionnaire that included items about the frequency of consumption of beverages (coffee, green tea, black tea) and food items, as well as alcohol drinking, smoking and other health-related lifestyle factors. We asked the subjects about their frequency of green-tea consumption according to five categories: never, occasionally, 1–2 cups per day, 3–4 cups per day and 5 or more cups per day. The validity of green-tea consumption was assessed by calculating Spearman correlation coefficients between the 12-day dietary records and the 40-item food-frequency questionnaire. The age- and energy-adjusted Spearman correlation coefficient in men was 0.71 (Ogawa *et al*, 2003). After exclusion of subjects with missing responses or with a prior history of cancer, 19 561 subjects remained. We followed up the vital and residential status of the subjects using population registries from 1 January 1995 to 31 December 2001. Reference to population-based cancer registries identified 110 incident cases of prostate cancer (7 years of follow-up with 121 543 person-years). During the study period, there was no mass screening programme for prostate cancer in this area.

We combined the lower two categories of green-tea consumption into the single category ‘less than one cup per day’ because of the small number of subjects in each category. We estimated hazard ratios (HRs) and the 95% confidence interval (CI) of prostate cancer incidence according to green-tea consumption, using the Cox proportional hazards model with adjustment for age and potential confounders. *P*-values for the test of linear trend were calculated by treating the green-tea consumption category as an ordinal variable. All *P*-values were two-tailed. This study had approximately 80% statistical power, with a two-sided *α*-error level of 5%, in detecting a true HR of 0.75 among the highest *vs* lowest categories of green-tea consumption.

## RESULTS

Table 1 shows the characteristics of the subjects according to green-tea consumption. Subjects with a higher green-tea intake tended to be older, to have a higher calorie intake, to consume calcium and fish more frequently, and to drink coffee less frequently.

We found no significant association between green-tea consumption and the risk of prostate cancer. Multivariate HRs for prostate cancer associated with drinking 1–2, 3–4 and 5 or more cups of green-tea per day, as compared with less than one cup per day, were 0.77 (95% CI 0.42–1.40), 1.15 (0.69–1.94), and 0.85 (0.50–1.43), respectively (trend *P*=0.81) (Table 2). Exclusion of prostate cancer cases diagnosed in the first 3 years of follow-up did not substantially change the results.

When the data were stratified according to age, smoking, alcohol drinking, body mass index, frequencies of meat and fish consumption, and frequencies of coffee and black tea consumption, there was no association between green-tea consumption and the risk of prostate cancer.

We also examined the relationship between consumption of black tea or coffee and the risk of prostate cancer. The multivariate HRs (95% CI) compared with men who never drank black tea were 1.34 (0.77–2.34) for those drinking black tea occasionally and 0.60 (0.13–2.68) for those drinking one or more cups per day (trend *P*=0.78). The corresponding HRs for coffee were 0.60 (0.35–1.05) and 0.67 (0.38–1.19) (trend *P*=0.27). Our results were consistent with the judgment of the World Cancer Research Fund that consumption of black tea or coffee has no relationship with the risk of prostate cancer (World Cancer Research Fund, 1997).

## DISCUSSION

This is the first prospective cohort study of green-tea consumption and prostate cancer incidence in Japan. We found no association between green-tea consumption and prostate cancer incidence among Japanese men, who consume green tea much more frequently than men in Western countries. Our results conflicted with those of a case–control study in China (Jian *et al*, 2004), but agreed with those of a prospective study in Hawaii and a case–control study in Japan showing no association between green-tea consumption and prostate cancer incidence (Severson *et al*, 1989; Sonoda *et al*, 2004).

Our study had several methodologic advantages over previous studies of the subject. We recruited subjects from the general population, and there was a large variation in green-tea consumption among our subjects. In addition, we assessed the consumption of green-tea and other variables before cases of prostate cancer were diagnosed, thus avoiding recall bias. The questionnaire used to measure green-tea consumption had a reasonably high level of validity and reproducibility.

As a potential limitation of the study, we could not specifically examine the effect of very high consumption of green tea because the highest category in our questionnaire was five or more cups per day. However, the validation study of our food frequency questionnaire found that 53% of the subjects who reported consuming five or more cups per day actually consumed seven or more cups per day according to 12-day diet records (Ogawa *et al*, 2003). It is therefore unlikely that we failed to detect a decreased risk of prostate cancer among the subjects consuming very large amounts of green tea.

In conclusion, this prospective cohort study conducted in rural Japan showed no association between consumption of green tea, coffee or black tea and the risk of prostate cancer.

## Figures and Tables

**Table 1:** Characteristics of the subjects according to green-tea consumption

	**Green-tea consumption (cups per day)**			
**Characteristic**	**<1**	**1 or 2**	**3 or 4**	**≥5**
No.	5982	4460	3397	5122
Age (years), means±s.d.	57.8±10.7	58.0±10.9	60.5±10.4	61.9±9.9
				
*Smoking* (%)				
Never	20.5	19.1	19.1	16.9
Past	24.7	24.0	27.5	27.8
Current	54.8	56.9	53.4	55.3
				
*Alcohol drinking* (%)				
Never	16.4	14.6	15.2	18.3
Past	10.5	9.8	10.2	11.5
Current	73.1	75.6	74.6	70.2
				
*Body mass index* (%)				
<18.5	8.4	7.6	6.4	7.6
18.5–24.9	65.3	67.0	68.6	67.7
≥25.0	26.3	25.4	25.0	24.7
				
Daily calorie intake (kcal day^−1^), means±s.d.	1776±614	1809±602	1847±589	1902±592
Daily calcium intake (mg day^−1^), means±s.d.	373±163	400±163	423±160	457±160
				
*Walking duration* (%)				
At least 1 h day^−1^	49.6	48.1	45.8	48.6
Under 1 h day^−1^	50.4	51.9	54.2	51.4
				
*Meat consumption* (%)				
Few	28.6	24.8	26.1	29.0
1–2 times/month	48.8	50.9	50.2	47.5
1–2 or more times/week	22.6	24.3	23.7	23.5
				
*Fish consumption* (%)				
Few or 1–2 times/week	35.5	31.7	26.8	22.1
3–4 times/week	32.6	34.8	36.4	33.8
Daily	31.9	33.5	36.8	44.1
				
*Coffee consumption* (%)				
Never	23.4	17.3	19.4	23.1
Occasionally	33.0	32.3	38.2	41.3
1–2	29.1	37.1	31.1	23.8
≥3	14.5	13.3	11.3	11.8
				
*Black tea consumption* (%)				
Never	68.8	63.4	63.2	66.3
Occasionally	29.1	30.6	32.6	30.2
1–2	1.7	5.3	2.5	2.1
≥3	0.4	0.7	1.7	1.4

*n*=19 561. s.d. denotes standard deviation.

**Table 2:** HRs and 95% CIs of prostate cancer according to green-tea consumption

	**Green-tea consumption (cups per day)**				
**Variable**	**<1**	**1 or 2**	**3 or 4**	**≥5**	**Trend *P***
No. of cases	29	18	31	32	
Person-years	36925	27658	24788	32172	
Age-adjusted HR	1.00	0.79 (0.44–1.43)	1.26 (0.76–2.09)	0.90 (0.55–1.50)	0.96
Multivariate HR^a^	1.00	0.77 (0.42–1.40)	1.15 (0.69–1.94)	0.85 (0.50–1.43)	0.81

HR=hazard ratio; CI=confidence interval.

a Mulitivariate HR was adjusted for age (in years), body mass index (<18.5, 18.5–24.9 and ≥25.0), alcohol consumption (never, former and current drinking), smoking status (never, former, and current smoking), marital status (marriage at age<25, 25–29, ≥30, unmarried, separated or divorced), daily calorie intake (continuous), daily calcium intake (tertile), walking duration (<1 h day^−1^, and ≥1 h day^−1^) , consumption frequencies of black tea and coffee (never, occasionally, 1–2, and >3 cups per day), consumption frequencies of meat (few, 1–2 times/month, 1–2 or more times/week) and consumption frequencies of fish (few or 1–2 times/week, 3–4 times/week, daily).
